# Bipolar At-Risk Criteria and Risk of Bipolar Disorder Over 10 or More Years

**DOI:** 10.1001/jamanetworkopen.2023.34078

**Published:** 2023-09-15

**Authors:** Aswin Ratheesh, Dylan Hammond, Michael Watson, Jennifer Betts, Emma Siegel, Patrick McGorry, Michael Berk, Susan Cotton, Andrew Chanen, Barnaby Nelson, Andreas Bechdolf

**Affiliations:** 1 Orygen, Parkville, Victoria, Australia; 2 Centre for Youth Mental Health, The University of Melbourne, Parkville, Victoria, Australia; 3 Institute for Mental and Physical Health and Clinical Translation, Deakin University School of Medicine, Barwon Health, Geelong, Victoria, Australia; 4 Department of Psychiatry, Psychotherapy and Psychosomatic Medicine, Vivantes Klinikum am Urban und Vivantes Klinikum im Friedrichshain, Berlin, Germany; 5 Department of Psychiatry and Psychotherapy, Campus Charité Mitte, Charité-Universitätsmedizin, Berlin, Germany

## Abstract

**Importance:**

Predicting the onset of bipolar disorder (BD) could facilitate preventive treatments. Among risk measures, bipolar at-risk (BAR) criteria have shown promise in predicting onset of bipolar disorder in the first year in clinical cohorts; however, it is not known whether BAR criteria are associated with the onset of BD in the longer term.

**Objective:**

To assess the association of BAR criteria with onset of BD over 10 to 13 years follow-up.

**Design, Setting, and Participants:**

This prospective cohort study, completed between May 1, 2020, and November 7, 2022, included consenting people seeking help for nonpsychotic major mental health difficulties, including mood, personality, and substance use disorders, who were originally recruited at ages 15 to 25 years from a tertiary youth mental health setting in metropolitan Melbourne, Victoria, Australia, from May 1, 2008, to September 30, 2010.

**Exposure:**

Meeting BAR criteria at baseline. Criteria included subthreshold mania, cyclothymic features, subthreshold depression, and family history of BD. A matched clinical comparison group was recruited from the same help-seeking population.

**Main Outcomes and Measures:**

The primary outcome was expert consensus diagnosis of BD I or II based on the Mini International Neuropsychiatric Interview, self-reported information collected through online assessments, and linked data on mental health service utilization in Victoria over 10 to 13 years of follow-up.

**Results:**

Among 69 eligible participants, follow-up data were available for 60 (88.2%). The mean (SD) age at the end of follow-up was 32.9 (2.8) years, and 49 (81.7%) were women. A total of 28 participants met BAR criteria, and 32 were in the comparison group. In the BAR group, 8 patients (28.6%) developed BD over a mean (SD) of 11.1 (0.7) years of follow-up, and no patients in the comparison group developed BD. The risk of developing BD was higher in the BAR group than in the non-BAR group (χ^2^_1_ = 70.0; *P* < .001). The proportions of transitions to BD were equal in the first and second halves of the follow-up period.

**Conclusions and relevance:**

In this cohort study of participants seeking care for mental health difficulties, patients meeting the BAR criteria were significantly more likely to transition to BD over a decade after ascertainment compared with patients not meeting the BAR criteria. The findings suggest that those meeting BAR criteria may benefit from longer-term monitoring and support. Evaluation of predictive properties in longer-term studies using a risk measure will help with implementation of BAR criteria in clinical settings.

## Key Points

**Question:**

Are bipolar at-risk (BAR) criteria associated with the onset of bipolar disorder (BD) over 10 or more years of follow-up?

**Findings:**

In this cohort study of 69 help-seeking participants aged 15 to 25 years, those identified to be at risk of BD using BAR criteria at baseline had a significantly higher risk of developing new-onset BD (types I or II) than a clinical comparison group that did not meet BAR criteria in 10 to 13 years of follow-up.

**Meaning:**

The findings suggest that help-seeking adolescents and young adults identified to be at risk using BAR criteria may benefit from longer-term monitoring and support.

## Teaser

This cohort study assesses whether patients meeting bipolar at-risk criteria have greater risk of developing bipolar disorder at 10 to 13 years of follow-up than patients not meeting the criteria.

## Introduction

Preventive and early interventions may help to improve outcomes for people at risk of bipolar disorder (BD),^1,2^ a recurrent condition prevalent in 2% to 3% of the population worldwide. To achieve prevention, it is necessary to predict those who might develop the disorder. Prospective studies on prediction have focused on individuals at high risk of BD due to a family history of BD^3,4^ or other characteristics in nonclinical samples.^5^ Among those seeking help in clinical settings, there are relatively few risk criteria or measures with evidence of prospective predictive validity.^6^ The bipolar at-risk (BAR) criteria (Table 1) showed promise, with 11.4% to 22.7% developing BD over 1 to 2 years.^7,8,9^ However, it is unclear whether meeting BAR criteria is also associated with the onset of BD over the medium to long term. This is important as transitions to BD can occur over decades,^10^ potentially providing a longer window for preventive interventions. Therefore, we aimed to evaluate the association between BAR criteria and the onset of BD I or II over 10 to 13 years in a clinical sample of help-seeking adolescents and young adults.

**Table 1.** Bipolar At-Risk Criteria

Criterion	Description
Age	15-25 y
Group 1: subthreshold mania	2-4 Consecutive days of abnormally and persistently elevated, expansive, or irritable mood with at least 2 of the following: (1) inflated self-esteem or grandiosity, (2) decreased need for sleep (eg, feels rested after only 3 h of sleep), (3) more talkative than usual or pressure to keep talking, (4) flight of ideas or subjective experience that thoughts are racing, (5) distractibility, and (6) increased goal-directed activity (socially, at work, or sexually) or psychomotor agitation
Group 2: depression and cyclothymic features	Depression defined as at least 1 week of depressed mood or loss of interest or pleasure with at least 2 of the following: (1) significant weight loss, (2) insomnia or hypersomnia nearly every day, (3) psychomotor retardation or agitation, (4) fatigue or loss of energy, (5) feelings of worthlessness or excessive or inappropriate guilt, (6) diminished ability to think or concentrate, and (7) recurrent thoughts of death and/or recurrent suicidal ideation; cyclothymic features defined as numerous episodes with subthreshold manic symptoms not meeting group 1 criteria and numerous episodes with depressive symptoms
Group 3: depression and genetic risk	Depression same as for group 2; genetic risk defined as first-degree relative with bipolar disorder
Exclusion criteria	(1) History of manic episode of ≥4 d, (2) history of psychosis of ≥7 d, (3) past treatment with a mood stabilizer for ≥6 wk, (4) past treatment with an antipsychotic for ≥3 wk (15 mg per wk of haloperidol or equivalent), (5) IQ below the normal range, and (6) organic brain disorder

## Methods

This cohort study assessed participants originally included in a 12-month prospective study in Melbourne, Victoria, Australia.^9^ All participants provided informed consent to be contacted and have data used for future research. The present study was approved by The Royal Melbourne Hospital Human Research Ethics Committee and was completed between May 1, 2020, and November 7, 2022. The study followed the Strengthening the Reporting of Observational Studies in Epidemiology (STROBE) reporting guideline for cohort studies.

### Participants and Setting

Participants were originally recruited from Orygen, a tertiary youth mental health setting in Melbourne, from May 1, 2008, to September 30, 2010. Individuals aged 15 to 25 years seeking help for nonpsychotic major mental health difficulties, including mood, personality, and substance use disorders were included. The included participants have been described in a previous publication^9^ and in the eMethods in Supplement 1. One participant died during the original study. The remaining participants were recontacted using methods established in previous cohort studies conducted at our center.^11,12^ When able to be contacted, participants could consent to provide information through several means. When unable to be contacted, we obtained information on participants’ mental health diagnoses by linking health and administrative records.

The exposure was meeting BAR criteria at baseline. Criteria included subthreshold mania, cyclothymic features, subthreshold depression, and family history of BD. A matched clinical comparison group was recruited from the same help-seeking population.

### Measures and Outcomes

Demographic information was collected using a purpose-built measure used at our center in both interviews and self-report assessments. Gender categories included male, female, nonbinary, prefer not to say, and other. Ethnicity was obtained as a free text question in the original study but was ascertained as country of birth in the follow-up study.

All assessments were conducted via telephone or video interviews in compliance with COVID-19 restrictions. Trained research assistants with a bachelor’s or master’s degree in psychology conducted the assessments with regular supervision from an experienced psychiatrist (A.R.). Research assistants were blinded to participants’ original group assignment. All contacted participants were offered a structured diagnostic interview using the mood and psychosis modules of the Mini International Neuropsychiatric Interview for *Diagnostic and Statistical Manual of Mental Disorders* (Fifth Edition),^13^ along with several secondary measures (detailed in the eMethods in Supplement 1). Based on lived-experience consultant feedback, we gave participants the option to provide their diagnostic and treatment information through a shorter interview, self-report online assessments, or through linking their medical records. Linked data provided information on contacts with tertiary mental health services. Diagnostic information coded in these public mental health registers reflect those deemed to be significant at the time of the contact. Such information has previously been used to establish follow-up diagnosis in similar cohorts in Victoria, Australia.^11,12^

Our primary outcome was an expert consensus diagnosis of BD I or II over 10 to 13 years of follow-up. These diagnoses were established for all participants with data from self-reported, interview-based, and/or linked data using multi-stage consensus meetings (described in the eMethods in Supplement 1) involving experienced psychiatrists and clinical psychologists. Diagnoses were made conservatively with BD diagnosis considered only when there was evidence of the participant meeting full diagnostic criteria or when the diagnosis was made by a psychiatrist with supporting evidence of treatment or help seeking for this condition.

### Statistical Analysis

We compared (1) participants who were followed up with those who were not, (2) participants meeting BAR criteria with those not meeting BAR criteria at follow-up, and (3) participants who later developed BD with those who did not. Comparisons were made using the Mann-Whitney *U* test for continuous variables (eg, age) and the χ^2^ test or Fisher exact test for categorical variables, alongside standardized effect sizes (Pearson *r *>0.3 and Cramer *V *>0.2 indicated moderate differences). The threshold for statistical significance was set at 2-sided *P* = .05. We analyzed transitions to BD I or II among the BAR and non-BAR groups using Kaplan-Meier survival curves. While the sample size was limited to those included in the original study, we expected this sample to have 80% power to detect a moderate to large effect size (*w *>0.4) in the difference in proportions developing BD at follow-up, accounting for a 20% attrition rate. Analysis was performed using R, version 4.3.0 (R Foundation for Statistical Computing).

## Results

Among participants included in the original study, 35 met BAR criteria (considered to be at risk of BD) and 35 did not (matched clinical comparison group). One participant died during the original study. Among 69 eligible participants, follow-up data were available for 60 (88.2%). Among these 60 participants (eFigure in Supplement 1), 49 (81.7%) were women, 10 (16.7%) were men, and 1 (1.6%) was nonbinary; the mean (SD) age at the end of follow-up was 32.9 (2.8) years. A total of 52 participants (86.7%) reported Australia as their country of birth, and the remaining participants reported having been born in India, Sri Lanka, Malaysia, Eritrea, Indonesia, and Vietnam. There were no meaningful demographic or clinical differences between those who were followed up and those who were not (eTable 1 in Supplement 1) except for women or nonbinary participants being more likely to be able to provide follow-up data than to not be able provide these data (50 [83.3%] vs 6 [10%]; Cramer *V* = 0.20). Of those followed up, BAR (n = 28) and non-BAR (n = 32) groups demonstrated moderate effect size differences in completion of tertiary education, likelihood of major depression diagnosis, and treatments received (Table 2 and eTable 2 in Supplement 1).

**Table 2.** Clinical, Demographic, and Treatment Characteristics of Participants Over the Follow-Up Period

Characteristic	Participants, No/total No. (%)		χ^2^	*P* value	Cramer *V*
	BAR (n = 28)	Non-BAR (n = 32)			
Age at end of follow-up, mean (SD), y	33.27 (3.06)	32.50 (2.71)	530.0^a^	.33	−0.12^b^
Gender					
Female	24/28 (85.7)	25/32 (78.1)	NA^c^	.67	0.10
Male	4/28 (14.3)	6/32 (18.8)	NA	NA	NA
Nonbinary	0	1/32 (3.1)	NA	NA	NA
Country of birth					
Australian	25/28 (89.3)	27/32 (84.4)	NA^c^	.86	0.07
Other					
All	3/28 (10.7)	5/32 (15.6)	NA	NA	NA
Eritrea	0	1/32 (3.1)	NA	NA	NA
India	2/28 (7.1)	0	NA	NA	NA
Indonesia	0	1/32 (3.1)	NA	NA	NA
Malaysia	1/28 (3.6)	0	NA	NA	NA
Sri Lanka	0	2/32 (6.3)	NA	NA	NA
Vietnam	0	1/32 (3.1)	NA	NA	NA
In a relationship	8/14 (57.1)	8/13 (61.5)	2.38	.67	0.04
Completed tertiary education	7/11 (63.6)	3/9 (33.3)	NA^c^	.37	0.30
Currently unemployed	2/13 (13.4)	3/14 (21.43)	NA^c^	.64	0.08
Diagnosis					
MINI					
Major depressive disorder, lifetime	8/12 (75.0)	14/14 (100)	NA^c^	.03	0.39
Psychotic symptoms or disorder	4/12 (25.0)	2/14 (14.3)	NA^c^	.64	0.14
Linkage data^d^					
Depressive disorder	20/25 (80.0)	26/30 (86.7)	0.44	.51	0.09
Borderline personality disorder	14/25 (56.0)	13/30 (43.3)	0.88	.35	0.13
Other personality disorder	7/25 (28.0)	5/30 (16.7)	1.03	.31	0.14
Anxiety disorder	9/25 (36.0)	12/30 (40.0)	0.09	.76	0.04
Stress-related disorder	9/25 (36.0)	9/30 (30.0)	0.22	.64	0.06
Substance use–related diagnosis^e^	10/25 (40.0)	7/30 (23.3)	1.77	.18	0.18
Treatment					
Counselling or therapy	11/13 (84.6)	11/14 (78.6)	NA^c^	>.99^f^	0.08
Mood stabilizer prescribed	6/11 (54.6)	2/11 (18.2)	NA^c^	.18^f^	0.38
Other medication prescribed	11/13 (84.6)	11/11 (100)	NA^c^	.48	0.28
Engaged with tertiary mental health care service	7/13 (23.1)	2/14 (28.6)	NA	>.99^f^	0.11
Engaged with psychologist	12/13 (92.3)	10/14 (71.4)	NA	.33	0.30

Abbreviations: MINI, Mini International Neuropsychiatric Interview; NA, not applicable.

^a^ Mann-Whitney *U* statistic.

^b^ Pearson *r*.

^c^ Fisher exact test used.

^d^ Any diagnosis recorded.

^e^ Other than tobacco.

^f^ Two-sided Fisher exact test performed when the expected cell number was less than 5.

Among patients meeting BAR criteria, 8 (28.6%) transitioned to BD over a mean (SD) of 11.1 (0.7) years of follow-up. No patients in the comparison group developed BD by the end of follow-up. Those meeting BAR criteria were significantly more likely than those not meeting BAR criteria to develop BD I or II by the end of follow-up (χ^2^_1_ = 70.0; *P* < .001). Equal numbers of transitions occurred in the first and second halves of the follow-up period (Figure). Seven transitions (87.5%) were to BD II and 1 (12.5%) to BD I. The source of the final BD diagnosis was most commonly from diagnostic interview (5 [62.5%]) (eTable 3 in Supplement 1). With respect to baseline BAR subgroups, 6 transitions (75.0%) occurred in those with subthreshold mania and 2 (25.0%) in those with major depression and cyclothymic features. Among baseline clinical or demographic characteristics, only higher manic symptom severity was associated with later onset of BD (eTable 4 in Supplement 1).

**Figure.**
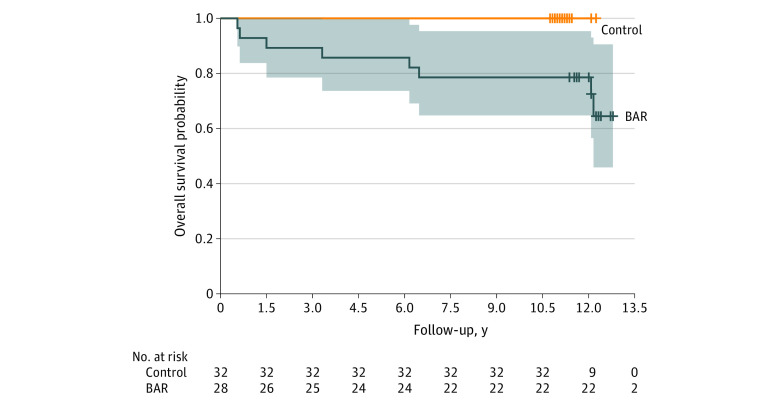
Kaplan-Meier Survival Curves for Participants Who Did and Did Not Meet Bipolar At-Risk (BAR) Criteria Shaded area indicates 95% CI; hash marks indicate right censoring.

## Discussion

In this, to our knowledge, first long-term (>10 years) cohort study of help-seeking participants at risk of BD using a priori risk criteria, 28.6% of those meeting BAR criteria developed BD by the end of 10- to 13-year follow-up. This finding suggests that BAR criteria may of have value in predicting BD among help-seeking adolescents and young adults. The observation that transitions continued to occur throughout the follow-up period indicates that the window for providing enhanced support, monitoring, or preventive interventions may extend into the third and fourth decades of life.

### Limitations

One limitation of this study is that we may have underestimated BD onset as data linkage did not include contacts with private psychiatrists and those with more severe illness may be less likely to be followed up.^14^ A total of 44 participants (62.8%) in the eligible sample did not have a structured diagnostic assessment, and the small sample size prevented adjustment of confounders. The relatively small, predominantly female sample referred to a tertiary youth mental health clinic may also limit generalizability to other populations. However, acceptable clinical utility indexes in other samples^15^ suggest that BAR criteria may be useful for screening and case finding in clinical youth mental health settings. This is supported by the availability of a semistructured measure targeting expanded BAR criteria with reasonable diagnostic accuracy.^8^

## Conclusions

In this cohort study of participants seeking care for mental health difficulties, patients meeting the BAR criteria were significantly more likely to transition to BD over a decade after ascertainment compared with patients not meeting the BAR criteria. To implement BAR criteria clinically, there is an urgent need for assessment of long-term predictive properties and case finding accuracy, which could pave the way for preventive intervention trials.

## Notes

Supplement 1.
eMethods.

eReferences.
**eFigure.** Flow diagram of included participants **eTable 1.** Associations between baseline clinical and demographic characteristics and whether participants were followed up **eTable 2.** Cross-sectional measures obtained from participants at the follow-up time point **eTable 3.** BD diagnosis at follow-up and data sources providing information for consensus diagnoses **eTable 4.** Associations between baseline clinical and demographic characteristics and BD outcome at follow-up

Supplement 2.
Data Sharing Statement

## References

[1] Vieta E, Salagre E, Grande I, . Early intervention in bipolar disorder. Am J Psychiatry. 2018;175(5):411-426. doi:10.1176/appi.ajp.2017.17090972 29361850

[2] Ratheesh A, Cotton SM, Davey CG, . Ethical considerations in preventive interventions for bipolar disorder. Early Interv Psychiatry. 2017;11(2):104-112. doi:10.1111/eip.12340 27027848

[3] Duffy A. The early stages of bipolar disorder and recent developments in the understanding of its neurobiology. Future Neurol. 2010;5(2):317-323. doi:10.2217/fnl.10.3

[4] Hafeman DM, Merranko J, Goldstein TR, . Assessment of a person-level risk calculator to predict new-onset bipolar spectrum disorder in youth at familial risk. JAMA Psychiatry. 2017;74(8):841-847. doi:10.1001/jamapsychiatry.2017.1763 28678992 PMC5710639

[5] Kwapil TR, Miller MB, Zinser MC, Chapman LJ, Chapman J, Eckblad M. A longitudinal study of high scorers on the hypomanic personality scale. J Abnorm Psychol. 2000;109(2):222-226. doi:10.1037/0021-843X.109.2.222 10895560

[6] Ratheesh A, Berk M, Davey CG, McGorry PD, Cotton SM. Instruments that prospectively predict bipolar disorder—a systematic review. J Affect Disord. 2015;179:65-73. doi:10.1016/j.jad.2015.03.025 25845751

[7] Bechdolf A, Nelson B, Cotton SM, . A preliminary evaluation of the validity of at-risk criteria for bipolar disorders in help-seeking adolescents and young adults. J Affect Disord. 2010;127(1-3):316-320. doi:10.1016/j.jad.2010.06.016 20619465

[8] Fusar-Poli P, De Micheli A, Rocchetti M, . Semistructured Interview for Bipolar At Risk States (SIBARS). Psychiatry Res. 2018;264:302-309. doi:10.1016/j.psychres.2018.03.074 29665559

[9] Bechdolf A, Ratheesh A, Cotton SM, . The predictive validity of bipolar at-risk (prodromal) criteria in help-seeking adolescents and young adults: a prospective study. Bipolar Disord. 2014;16(5):493-504. doi:10.1111/bdi.12205 24797824

[10] Angst J, Sellaro R, Stassen HH, Gamma A. Diagnostic conversion from depression to bipolar disorders: results of a long-term prospective study of hospital admissions. J Affect Disord. 2005;84(2-3):149-157. doi:10.1016/S0165-0327(03)00195-2 15708412

[11] Nelson B, Yuen HP, Wood SJ, . Long-term follow-up of a group at ultra high risk (“prodromal”) for psychosis: the PACE 400 study. JAMA Psychiatry. 2013;70(8):793-802. doi:10.1001/jamapsychiatry.2013.1270 23739772

[12] Cotton S, Filia K, Watson A, . A protocol for the first episode psychosis outcome study (FEPOS): ≥15 year follow-up after treatment at the Early Psychosis Prevention and Intervention Centre, Melbourne, Australia. Early Interv Psychiatry. 2022;16(7):715-723. doi:10.1111/eip.13204 34415106

[13] Sheehan DV, Lecrubier Y, Sheehan KH, . The Mini-International Neuropsychiatric Interview (M.I.N.I.): the development and validation of a structured diagnostic psychiatric interview for *DSM-IV* and *ICD-10*. J Clin Psychiatry. 1998;59(suppl 20):22-33. 9881538

[14] Allott K, Chanen A, Yuen HP. Attrition bias in longitudinal research involving adolescent psychiatric outpatients. J Nerv Ment Dis. 2006;194(12):958-961. doi:10.1097/01.nmd.0000243761.52104.91 17164636

[15] Scott J, Marwaha S, Ratheesh A, . Bipolar at-risk criteria: an examination of which clinical features have optimal utility for identifying youth at risk of early transition from depression to bipolar disorders. Schizophr Bull. 2017;43(4):737-744. doi:10.1093/schbul/sbw154 27872258 PMC5472157

