# Supplementary material for: Bipolar At-Risk Criteria and Risk of Bipolar Disorder Over 10 or More Years
Source: JAMA Netw Open. 2023 Sep 15;6(9):e2334078. doi: 10.1001/jamanetworkopen.2023.34078 (PMC10504610; doi:10.1001/jamanetworkopen.2023.34078)
Supplement: Supplement 1. — eMethods. eReferences. eFigure. Flow diagram of included participants eTable 1. Associations between baseline clinical and demographic characteristics and whether participants were followed up eTable 2. Cross-sectional measures obtained from participants at the follow-up time point eTable 3. BD diagnosis at follow-up and data sources providing information for consensus diagnoses eTable 4. Associations between baseline clinical and demographic characteristics and BD outcome at follow-up [file jamanetwopen-e2334078-s001.pdf]

Supplemental Online Content

Ratheesh A, Hammond D, Watson M, et al. Bipolar at-risk criteria and risk of bipolar disorder. *JAMA Netw Open.* 2023;6(9):e2334078. doi:10.1001/jamanetworkopen.2023.34078

eMethods.

eReferences.

eFigure. Flow diagram of included participants

eTable 1. Associations between baseline clinical and demographic characteristics and whether participants were followed up

eTable 2. Cross-sectional measures obtained from participants at the follow-up time-point

eTable 3. BD diagnosis at follow up and data sources providing information for consensus diagnoses

eTable 4. Associations between baseline clinical and demographic characteristics and BD outcome at follow up

This supplemental material has been provided by the authors to give readers additional information about their work.

© 2023 Ratheesh A et al. *JAMA Network Open.*

eMethods

Setting

This study and the original BARPS study[1] were conducted at Orygen, a tertiary youth mental health service in Melbourne, Australia. Referrals to Orygen are taken from a range of sources including general practitioners and other primary care services, educational support services, drug and alcohol services, carers, families and young people themselves. Triage clinicians perform clinical assessments and refer patients to the respective teams.

Youth and lived experience consultation

As part of the routine procedures for local ethical approvals at Orygen, we sought feedback on the study design from Youth Research Committee representatives with lived experience of mental ill health including BD. This led to modification of the proposal to allow participants to provide follow-up information through a diagnostic interview, or through shorter interviewer-rated or self-report assessments, or finally via data-linkage.

Participants

Participants were recruited for this study if they participated in the original BARPS study.[1] At recruitment for the initial study, all 70 participants consented to participate in future research and 69 consented to allow use of research data or their medical records for future contact and research purposes. Only participants with sufficient data to determine mental health diagnoses were included in the current report.

In the original BARPS study, participants were selected for the BAR group if they were aged 15 to 24 years, and were found to have sub-threshold manic symptoms, depression with cyclothymic features, or depression with genetic risk for BD. The individual sub-criteria that constitute BAR criteria were established using a combination of direct interviews with patients, medical history as assessed by triage or treating clinicians. The inclusions were confirmed by a research assistant (JB) and confirmed with the principal investigator of the original study (AB). The non-BAR control group did not meet the BAR criteria and were group-matched by gender, age, previous hospitalisation, and use of antidepressants at intake. Participants were excluded if they had prior or current episodes of (hypo)mania lasting four or more days or psychosis lasting seven or more days; reported previous use of a mood stabiliser for more than six weeks, use of 100 mg per day or more of quetiapine or an equivalent atypical antipsychotic, or use of 100 mg per day or more chlorpromazine or an

© 2023 Ratheesh A et al. *JAMA Network Open.*

equivalent typical antipsychotic for over 28 days; IQ below normal range; or an organic brain disorder.

Selection of original participants: A research assistant (JB) screened intake assessments at Orygen to identify patients who were eligible for the BAR and non-BAR groups. The research assistant consulted regularly with triage and treating clinicians to identify clients that may meet the study's inclusion criteria. Clients who met eligibility were approached by the research assistant and invited to participate in the study. The study aimed to recruit 70 participants and recruitment was continued till the same was achieved. Recruitment took place from May 2008 until September 2010. A total of 559 help seeking adolescents and young adults were screened. Of these 172 patients met exclusion criteria. 59 patients (10.6% of the overall sample) fulfilled BAR criteria and no exclusion criteria. Of these 59 patients, 18 refused to participate and 6 could not be contacted. Thus, 35 of 59 eligible patients (59.3%) were recruited into the study. Of the 35 included BAR patients, one patient died of suicide. Of the patients without exclusion criteria, 46 of those who matched participants in the BAR group were approached. Of those, 11 did not give informed consent to the study or could not be contacted. Thus, 35 non-BAR patients were recruited into the study.

Description of sample at baseline

At baseline, the BAR group mainly comprised patients meeting the criteria for depression and cyclothymic features (Group II, 57.2 %, n=20), followed by the sub-threshold mania (Group I, 28.6%, n=10), and then depression and genetic risk (Group III, 4.3%, n=3). Two patients met both group I and II criteria (5.7 %). The majority of participants were female, were single, were living with family members, were students or unemployed, and were born in Australia. There were no significant differences between the BAR and control groups on any of the demographic variables. Further details are available in a previous publication.[1]

At baseline, the sample included a quarter of those who scored above the recommended cut-off for borderline personality pathology. This is likely related to the fact that youth seeking help at a tertiary youth mental health service for non-psychotic diagnoses (our eligible population) are likely to have significant personality pathology, given the association between self-harm, risk taking and referral for ongoing care. This is consistent with data from other centres where 33% of hospitalised youth met criteria for BPD at intake.[2]

All participants provided consent to be contacted for future research. All except one, provided consent to have their health records accessed for this study.

© 2023 Ratheesh A et al. *JAMA Network Open.*

Procedures in current study

The procedures to contact participants were consistent with our previous follow up studies.[3, 4] In order to avoid distress to family members and significant others on follow-up contact, the National Death Index was used to determine if any participants had died following the initial study. Following this, all participants were sent a letter inviting them to participate using the contact details provided during the initial study. Additional efforts were made to contact participants including alternative phone numbers, addresses, and next of kin details. Failing this, online search methods such as Google and social media were used to access publicly available contact information for participants. If such information could not be identified by these means, health records (which participants had provided consent to access) were searched in order to obtain updated details.

When participants were able to be contacted, informed consent was obtained through a form signed before a witness or online via a secure telehealth platform. In light of COVID-19 restrictions, telehealth interviews for consenting and assessments were offered to all participants to provide informed consent, complete the assessments, and discuss referral needs. Given the long period since original contact and based on initial feedback from our lived experience consultants, it was considered that some participants may not be interested in or be able to tolerate a detailed interview on follow up. These participants were therefore given the option to provide their diagnostic and treatment information through a short interview, self-report online assessments, or through consent for linking their medical records.

All assessments were performed by research assistants with at least a bachelor-level qualification in psychology. Participants were reimbursed for their time consistent with local and ethically approved procedures.

Measures and data sources

a) Full interview: The full assessment consisted of a core assessment along with additional components. The elements of the core assessment were participant demographics, current and past mental health diagnoses and treatments and current functioning using the Social and Occupational Functioning Assessment Scale (SOFAS).[5] Additional elements of the full interview included the mood and psychotic

© 2023 Ratheesh A et al. *JAMA Network Open.*

disorders modules of the Mini International Neuropsychiatric Interview (MINI) [6] and substance use using the World Health Organisation Alcohol, Smoking, and Substance Involvement Screening Test (WHO ASSIST).[7] Participants were also invited to complete a self-reported assessment online with further demographic information, diagnostic information (Altman Self-Rating Scale for Mania,[8] Patient Health Questionnaire 8,[9] General Anxiety Disorder-7),[10] quality of life (Assessment of Quality-of-Life 4D),[11] personality functioning (Personality Inventory for DSM-5 – Brief Form).[12] Demographic information was collected using a purpose-built measure utilised at our centre, in both interviews and self-report assessments. Gender categories included male, female, non-binary, prefer not to say and other. We also obtained participants' country of birth.

b) Abbreviated interview: The core assessment elements including participant demographics, current and past mental health diagnoses and treatments, and current functioning using SOFAS.

c) Data linkage: Data were obtained regarding participants' contacts with the Victorian Mental Health register (CMI) or the register for admitted hospital episodes (Victorian Admitted Episodes Dataset, VAED). CMI is a state-wide mental health register that documents contact details of individuals based on their most recent contact with the Victorian health system. This includes emergency department (ED) contacts, contacts during inpatient admissions, and community-based case management support. VAED provides a comprehensive dataset of the causes, effects and nature of illness, and the use of health services in Victoria. All linkage occurred through the Centre for Victorian Data Linkage.

Outcomes:

The primary outcome of interest for this study was an expert consensus diagnosis of BD, determined across the Mini International Neuropsychiatric Interview (MINI), a self-report of diagnosis of BD-I or -II by a qualified clinician and documented evidence of BD diagnosis using linkage data.

Secondary outcomes included functional outcomes, their relationship with the BAR criteria and other baseline characteristics; qualitative reports of treatment received; symptomatic status, substance use, quality of life, personality functioning, and comorbid mental illnesses.

© 2023 Ratheesh A et al. *JAMA Network Open.*

Other linkage diagnoses reported (Table 2) reflect any diagnoses made using MINI or from data linkage and do not represent consensus diagnoses.

Consensus procedure

First, the information collected by RAs at follow up was reviewed with a trained psychiatrist (RA) and psychologist (DH) and diagnoses were established. Senior clinical psychiatrists or psychologists (AC and BN) adjudicated on ambiguities or discrepant diagnoses between data sources. All raters were blinded to participants' original group assignment at this stage. Second, these diagnoses were linked to the participants' one-year follow up diagnoses. Further consensus steps were undertaken to resolve discrepancies as outlined above.

Principles of diagnostic ascertainment in consensus meetings

1. The best estimate of follow up diagnoses at 10-13 years, were estimated from the following sources of information, in their hierarchical order of reliability.
    a. Diagnoses based on MINI were considered gold-standard whenever available. When MINI information was insufficient due to participant recall or other factors, other diagnostic information was discounted.
    b. In the absence of MINI data, self-report diagnoses of BD I or II were considered to be acceptable if this diagnosis was made by a psychiatrist or a tertiary mental health service.
    c. In the absence of both these sources of information, linkage results were considered acceptable if there were one or more instances of BD diagnoses ascertained by a tertiary mental health clinician during outpatient mental health care, inpatient admission, or during an ED visit.
2. Integrating one-year and 10-13 year follow up assessments: When a BD diagnosis had been made at one-year assessment but not at 10-13 year follow up, all relevant information was considered to arrive at a final diagnosis.
    a. Diagnostic information ascertained at 10-13 years was considered more likely to represent the longer-term diagnostic picture, compared with the diagnoses made at one year.
    b. Year one diagnoses were discounted if 10-13-year diagnosis of BD could not be established based on treatment information, mental health help-seeking, or the number of threshold symptoms reported on MINI interviews.

© 2023 Ratheesh A et al. *JAMA Network Open.*

eReferences

1. Bechdolf A, Ratheesh A, Cotton SM, et al. The predictive validity of bipolar at-risk (prodromal) criteria in help-seeking adolescents and young adults: a prospective study. *Bipolar Disord*. Aug 2014;16(5):493-504. doi:10.1111/bdi.12205
2. Ha C, Balderas JC, Zanarini MC, Oldham J, Sharp C. Psychiatric comorbidity in hospitalized adolescents with borderline personality disorder. *J Clin Psychiatry*. May 2014;75(5):e457-64. doi:10.4088/JCP.13m08696
3. Nelson B, Yuen HP, Wood SJ, et al. Long-term follow-up of a group at ultra high risk ("prodromal") for psychosis: the PACE 400 study. *JAMA Psychiatry*. Aug 2013;70(8):793-802. doi:1695587 [pii]
10.1001/jamapsychiatry.2013.1270
4. Cotton S, Filia K, Watson A, et al. A protocol for the first episode psychosis outcome study (FEPOS): >/=15 year follow-up after treatment at the Early Psychosis Prevention and Intervention Centre, Melbourne, Australia. *Early Interv Psychiatry*. Jul 2022;16(7):715-723. doi:10.1111/eip.13204
5. American Psychiatric Association. *Diagnostic and statistical manual of mental disorders (4th edition- Text revision)*. American Psychiatric Publishing; 2000.
6. Sheehan DV, Lecrubier Y, Sheehan KH, et al. The Mini-International Neuropsychiatric Interview (M.I.N.I.): the development and validation of a structured diagnostic psychiatric interview for DSM-IV and ICD-10. *J Clin Psychiatry*. 1998;59 Suppl 20:22-33;quiz 34-57.
7. Who Assist Working Group. The Alcohol, Smoking and Substance Involvement Screening Test (ASSIST): development, reliability and feasibility. *Addiction*. Sep 2002;97(9):1183-94.
8. Altman EG, Hedeker D, Peterson JL, Davis JM. The Altman Self-Rating Mania Scale. *Biol Psychiatry*. Nov 15 1997;42(10):948-55. doi:S0006-3223(96)00548-3 [pii]
10.1016/S0006-3223(96)00548-3 [doi]
9. Kroenke K, Strine TW, Spitzer RL, Williams JB, Berry JT, Mokdad AH. The PHQ-8 as a measure of current depression in the general population. *J Affect Disord*. Apr 2009;114(1-3):163-73. doi:10.1016/j.jad.2008.06.026
10. Spitzer RL, Kroenke K, Williams JB, Lowe B. A brief measure for assessing generalized anxiety disorder: the GAD-7. *Arch Intern Med*. May 22 2006;166(10):1092-7. doi:10.1001/archinte.166.10.1092
11. Hawthorne G, Osborne R. Population norms and meaningful differences for the Assessment of Quality of Life (AQoL) measure. *Aust N Z J Public Health*. Apr 2005;29(2):136-42. doi:10.1111/j.1467-842x.2005.tb00063.x
12. American Psychiatric Association. The Personality Inventory for DSM-5—Brief Form (PID-5-BF)—Adult. 2023. https://www.psychiatry.org/File%20Library/Psychiatrists/Practice/DSM/APA_DSM5_The-Personality-Inventory-For-DSM-5-Brief-Form-Adult.pdf

© 2023 Ratheesh A et al. *JAMA Network Open.*

Supplementary Results

eFigure: Flow diagram of included participants.

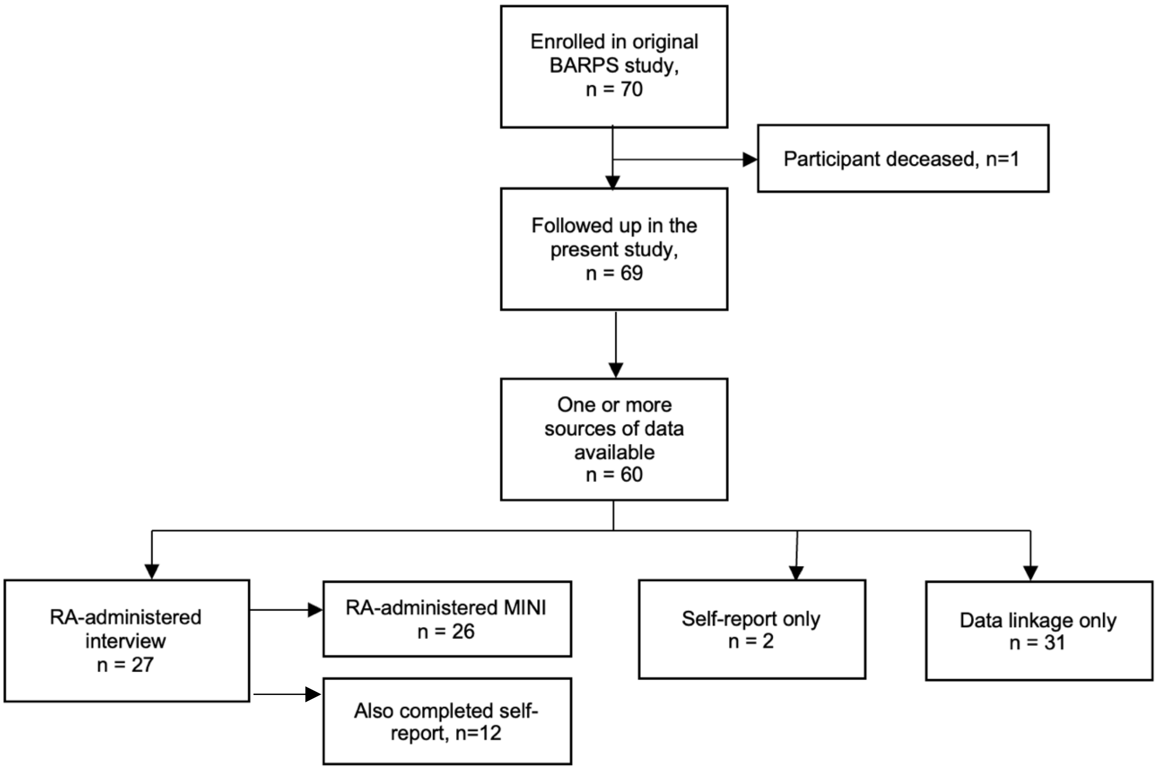

BARPS: Bipolar At-Risk Prospective Study, RA: Research Assistant; MINI: Mini International Neuropsychiatric Interview

© 2023 Ratheesh A et al. *JAMA Network Open.*

eTable 1: Associations between baseline clinical and demographic characteristics and whether participants were followed up

Characteristic	Followed up		Not followed up		Test	P-value
	Mean ± SD; Percent (count)	n	Mean ± SD; Percent (count)	n		
Demographics						
Age (years)	32.88 ± 2.77	60	32.90 ± 3.74	10	286.00[a]	0.814
Gender						
Female	81.67% (49)		60% (6)		FET	.20
Male	16.67% (10)		40% (4)			
Non-Binary	1.67% (1)		-			
Country of Birth						
Australia	86.67% (52)	60	90% (9)	10	FET	1.00
Other	13.33% (8)	60	10% (1)	10		
Eritrea	1.67% (1)	60	-	10		
India	3.33% (2)	60	-	10		
Indonesia	1.67% (1)	60	-	10		
Malaysia	1.67% (1)	60	-	10		
Sri Lanka	3.33% (2)	60	-	10		
Vietnam	1.67% (1)	60	-	10		
England	-	60	10% (1)	10		
In a relationship	38.33% (23)	60	40.00% (4)	10	FET	1.00
Living alone	5.00% (3)	60	0.00% (0)	10	FET	1.00
Employed	55.17% (32)	58	70.00% (7)	10	FET	0.494
Family history						
Depression	82.35% (42)	51	87.50% (7)	8	FET	1.00
Bipolar disorder	6.67% (4)	60	0.00% (0)	10	FET	1.00
Psychosis	17.65% (9)	51	37.50% (3)	8	FET	0.340
Clinical measures						
At Risk Mental State	48.15% (13)	27	71.43% (5)	7	FET	0.405
BPQ	44.09 ± 13.55	57	47.86 ± 13.11	7	169.00[a]	0.512
MADRS	19.82 ± 11.09	60	21.93 ± 9.97	10	268.00[a]	0.591
YMRS	8.69 ± 6.88	58	11.37 ± 6.89	9	196.00[a]	0.231
Treatment received at baseline						
Any medication	66.07% (37)	56	75.00% (6)	8	FET	0.711
Antidepressant medication	60.71% (34)	56	55.56% (5)	9	FET	1.00
Case management	25.00% (11)	44	16.67% (1)	6	FET	1.00
Bipolar variables						
Met BAR criteria	80.00% (28)	35	70.00% (7)	10	FET -	0.668

[a]Mann-Whitney U
FET: Fischer’s Exact Test

© 2023 Ratheesh A et al. *JAMA Network Open.*

BPQ: Borderline Personality Questionnaire
MADRS: Montgomery-Asberg Depression Rating Scale
YMRS: Youth Mania Rating Scale

© 2023 Ratheesh A et al. *JAMA Network Open.*

eTable 2: Cross-sectional measures obtained from participants at the follow-up time-point

Clinical measures							
Measure	BAR Median (Range)	n	Non-BAR Median (Range)	n	Mann-Whitney U	Effect size *r*	P-value
AQOL-4D	15.00 (19.00)	10	17.50 (13.00)	6	15.00	**-0.409**	0.101
ASRM	0.50 (13.00)	10	1.50 (4.00)	6	28.00	-0.058	0.816
GAD7	2.50 (20.00)	10	7.50 (17.00)	6	16.00	**-0.382**	0.126
SOFAS	75.00 (41.00)	13	71.00 (50.00)	14	86.50	-0.042	0.827
PID5 Total	7.00 (37.00)	10	17.00 (39.00)	6	19.00	-0.299	0.232
PID5 Negative Affect	1.50 (9.00)	10	5.00 (9.00)	6	15.50	**-0.396**	0.113
PID5 Detachment	2.00 (14.00)	10	5.00 (10.00)	6	20.00	-0.274	0.272
PID5 Antagonism	0.00 (2.00)	10	0.00 (4.00)	6	28.00	-0.062	0.803
PID5 Disinhibition	0.00 (4.00)	10	2.00 (14.00)	6	18.00	**-0.349**	0.163
PHQ-8 Total	4.00 (19.00)	7	5.00 (24.00)	6	17.00	-0.161	0.562
ASSIST Tobacco Risk	2.00 (1.00)	12	2.00 (2.00)	13	62.50	-0.191	0.339
ASSIST Alcohol Risk	1.00 (2.00)	12	1.00 (2.00)	13	76.00	-0.027	0.894
ASSIST Cannabis Risk	2.00 (1.00)	12	1.00 (2.00)	13	54.00	**-0.304**	0.129
ASSIST Cocaine Risk	1.00 (1.00)	12	1.00 (1.00)	13	77.50	-0.012	0.954

AQOL-4D: Assessment of Quality-of-Life version 4D
ASRM: Altman Self-Rating Mania Scale
GAD-7: Generalised Anxiety Disorder 7-item
SOFAS: Social and Occupational Functioning Assessment Scale
PID5: Personality Inventory for DSM-V
PHQ8: Patient Health Questionnaire version 8
ASSIST: Alcohol, Smoking and Substance Involvement Screening Test

© 2023 Ratheesh A et al. *JAMA Network Open.*

eTable 3: BD diagnosis at follow up and data sources providing information for consensus diagnoses

Final diagnoses based on all data		Data availability across time points and sources			
		12-month follow up	10-13 year follow up		
Consensus diagnosis between 1-13 years of follow up	**N**	**LIFE**	**MINI**	**Online self-reports**	**Data linkage**
Bipolar I	1	0	0	0	1
Bipolar II	7	2	5	4	6
Schizoaffective	1	0	0	0	1
Subthreshold BD	3	0	3	1	3
No BD	48	2	18	12	44

BD: Bipolar disorder
LIFE: Longitudinal Interval Follow-up Evaluation
MINI: Mini International Neuropsychiatric Interview

Note: Out of four participants with a 12-month diagnosis of BD II, two were re-interviewed using the MINI where they reported symptoms or functional change indicating that they were sub-threshold for BD. Therefore, they were considered not to have a long-term diagnosis of BD.

© 2023 Ratheesh A et al. *JAMA Network Open.*

eTable 4: Associations between baseline clinical and demographic characteristics and BD outcome at follow up

Variable	Developed BD		Did not develop BD		Test	P-value
	Mean ± SD; Percent (count)	n	Mean ± SD; Percent (count)	n		
Demographics						
Age	34.24 ± 2.83	8	32.68 ± 2.73	52	137.00[a]	0.123
Gender						
Female	87.50% (7)	8	80.77% (42)	52	FET	1.00
Male	12.50% (1)	8	17.31% (9)	52		
Non-Binary	-	8	1.92 % (1)	52		
Country of Birth						
Australia	87.50% (7)	8	86.54% (45)	52	FET	1.00
Other	12.50% (1)	8	13.46% (7)	52		
Eritrea	-	8	1.92% (1)	52		
India	12.50% (1)	8	1.92% (1)	52		
Indonesia	-	8	1.92% (1)	52		
Malaysia	-	8	1.92% (1)	52		
Sri Lanka	-	8	3.85% (2)	52		
Vietnam	-	8	1.92% (1)	52		
University qualification	80.00% (4)	5	40.00% (6)	15	FET	0.303
Family history						
Bipolar disorder	16.67% (1)	6	17.78% (8)	45	FET	1.00
Depression	83.33% (5)	6	82.22% (37)	45	FET	1.00
Psychosis	16.67% (1)	6	17.78% (8)	45	FET	1.00
Comorbid mental illness						
Depression	37.50% (3)	8	53.06% (26)	49	0.735[b]	0.693
Anxiety	62.50% (5)	8	61.22% (30)	49	0.096[b]	0.953
Substance use disorder	37.50% (3)	8	22.45% (11)	49	4.402[b]	0.111
Attenuated psychosis syndrome	0.00% (0)	4	56.52% (13)	23	FET	0.098
Symptom severity and functioning						
MADRS	22.25 ± 11.42	8	19.44 ± 11.11	52	175.00[a]	0.473

© 2023 Ratheesh A et al. *JAMA Network Open.*

YMRS	14.39 ± 7.33	8	7.78 ± 6.42	50	91.50[a]	**0.014**
BPQ	42.13 ± 14.00	8	44.41 ± 13.60	49	173.50[a]	0.605
SOFAS	68.50 ± 9.29	6	69.52 ± 14.57	21	51.50[a]	0.502
Treatment received						
Case management	42.86% (3)	7	21.62% (8)	37	FET	0.154
Antidepressant medication	87.50% (7)	8	56.25% (27)	48	FET	0.129
Any medication	87.50% (7)	8	62.50% (30)	48	FET	0.243

[a]Mann-Whitney U
[b]Pearson Chi-squared,
FET: Fischer’s Exact Test
BPQ: Borderline Personality Questionnaire
MADRS: Montgomery-Asberg Depression Rating Scale=
YMRS: Youth Mania Rating Scale
BD: Bipolar disorder
SOFAS: Social and Occupational Functioning Assessment Scale

© 2023 Ratheesh A et al. *JAMA Network Open.*
