# Supplementary material for: Bipolar At-Risk Criteria and Risk of Bipolar Disorder Over 10 or More Years
Source: JAMA Netw Open. 2023 Sep 15;6(9):e2334078. doi: 10.1001/jamanetworkopen.2023.34078 (PMC10504610; doi:10.1001/jamanetworkopen.2023.34078)
Supplement: Supplement 2. — Data Sharing Statement [file jamanetwopen-e2334078-s002.pdf]

Data Sharing Statement

Ratheesh. Bipolar At-Risk Criteria and Risk of Bipolar Disorder Over 10 or More Years. *JAMA Netw Open*. Published September 15, 2023. doi:10.1001/jamanetworkopen.2023.34078

Data
Data available: No
